# The association between workload, alcohol use, and alcohol misuse among psychiatrists in China

**DOI:** 10.3389/fpsyt.2023.1171316

**Published:** 2023-06-22

**Authors:** Wenzheng Li, Long Chen, Michael Hsu, Daming Mo, Lei Xia, Kaiyuan Min, Feng Jiang, Tingfang Liu, Yuanli Liu, Huanzhong Liu, Yi-lang Tang

**Affiliations:** ^1^Department of Psychiatry, Chao Hu Hospital of Anhui Medical University, Hefei, China; ^2^Hefei Fourth People’s Hospital, Hefei, China; ^3^Department of Psychiatry, School of Mental Health and Psychological Sciences, Anhui Medical University, Hefei, China; ^4^Affiliated Psychological Hospital of Anhui Medical University, Hefei, China; ^5^Addiction Psychiatry Fellowship Program, Department of Psychiatry and Behavioral Sciences, Emory University, Atlanta, GA, United States; ^6^Anhui Psychiatric Center, Hefei, China; ^7^State Key Laboratory of Medical Molecular Biology, Institute of Basic Medical Sciences, Chinese Academy of Medical Sciences and Peking Union Medical College, Beijing, China; ^8^School of International and Public Affairs, Shanghai Jiao Tong University, Shanghai, China; ^9^Institute of Healthy Yangtze River Delta, Shanghai Jiao Tong University, Shanghai, China; ^10^School of Health Policy and Management, Chinese Academy of Medical Sciences and Peking Union Medical College, Beijing, China; ^11^Atlanta Veterans Affairs Medical Center, Decatur, GA, United States

**Keywords:** alcohol use, alcohol misuse, psychiatrists, workload, association

## Abstract

**Aim:**

Survey alcohol use and workload among Chinese psychiatrists and explore their associations.

**Methods:**

We conducted an online questionnaire among psychiatrists working in large psychiatric institutions across the country. We collected data including demographic factors, alcohol use, and workload. Alcohol use was assessed using the Alcohol Use Disorder Identification Test-Consumption (AUDIT-C), and workload-related questions included working hours, night shifts, and caseloads.

**Results:**

In total, 3,549 psychiatrists completed the survey. Nearly half (47.6%) reported alcohol use, and the percentage of alcohol use in males (74.1%) was significantly higher than in females. 8.1% exceeded the AUDIT-C cutoff scores for probable alcohol misuse (19.6%in males and 2.6%in females). AUDIT-C scores were significantly correlated with working hours per week (*p* = 0.017) and the number of outpatient visits per week (*p* = 0.006). Regressional analysis showed that alcohol use was significantly associated with the following factors: longer working hours (Working more than 44 h/week, OR = 1.315), having an administrative position (OR = 1.352), being male (OR = 6.856), being single (OR = 1.601), being divorced or widowed (OR = 1.888), smoking (OR = 2.219), working in the West (OR = 1.511) or the Northeast (OR = 2.440). Regressional analysis showed that alcohol misuse was significantly associated with the following factors: fewer night shifts (Three to four night shifts/month, OR = 1.460; No more than 2 night shifts/month, OR = 1.864), being male (OR = 4.007), working in the Northeast (OR = 1.683), smoking (OR = 2.219), frequent insomnia (OR = 1.678).

**Conclusion:**

Nearly half of the psychiatrists in China reported alcohol use and 8.1% had probable AUD. Alcohol consumption is significantly associated with several workload-related factors, such as long working hours, heavy caseload, and administrative duties. Alcohol misuse was inversely associated with the number of night shifts per month. While the direction of causality is unclear, our findings may help identify vulnerable professional groups and develop more targeted interventions to improve healthcare professionals’ well-being.

## 1. Introduction

Alcohol is one of the leading causes of preventable death globally, with 3 million deaths each year directly or indirectly attributable to alcohol (1). In the United States, more than half of people over the age of 26 drink alcohol at least once a month, and more than a quarter of them drink alcohol to the point of binge drinking (2). In China, data released in 2019 showed a 12-month prevalence of alcohol use disorder of 1.8% and a lifetime prevalence of 4.4% (3). China is a country with a large production and consumption of alcohol. From World Health Organization (WHO) report, *per capita*, alcohol consumption in China has been increasing year by year, with an increase of 76% from 2005 to 2016 (1), the highest growth rate in the world (4).

Physicians are often viewed as societal role models and hence play an important role in health promotion and disease prevention *via* their own lifestyle habits (5), and may also play a crucial role in formulating health policy (6). Yet physicians, like the general population, can develop alcohol use disorder (AUD). Some studies showed that physicians are at a greater risk of developing AUD (7, 8), although the rates of AUD among physicians in different studies from different countries or regions vary greatly. An Austrian study found no significant difference in the rate of AUD between physicians (office-based physicians) and the general population, the same study suggests that doctors and geriatricians in rural areas may be at higher risk for AUD (9). A Belgian study showed that 18% of physicians could be classified as hazardous drinkers (10). A study from Germany showed that 20% of medical professionals had hazardous drinking (AUDIT-C scores of 5 or more for males and females) (11). A Norwegian study suggested that 21.7% of male and 7.6% of female specialists were hazardous drinkers (Alcohol use was measured using a modified version of the Alcohol Use Disorders Identification Test. A score of 9 or more was used as an indicator of hazardous drinking) (12). Findings from studies conducted in China also vary considerably. For example, a survey of 1,418 healthcare workers in general hospitals in Jiangsu Province in 2008 showed that the overall drinking rate of doctors was 14.0% (40.4% for men and 2.6% for women) (13). In 2019, a survey of mental health workers in China showed that the overall alcohol consumption rate was 41.8% (7.5% for probable AUD), with rates of 53.9% among psychiatrists (10.2% for probable AUD) and 36.2% among psychiatric nurses (6.3% for probable AUD) (14). Studies have shown that alcohol misuse or AUD often not only negatively impact physicians’ own health and well-being but also their professional ability and proneness to medical errors (15, 16).

On the other hand, healthcare professionals including doctors often face multiple job-related stressors, including heavy workloads (17), poor work-life balance (18), inadequate sleep (19), low pay levels, and contentious doctor-patient relationships and workplace violence (20). Among them, the relationship between physician workload and their well-being is a focus of concern (21). Workload is a generic term used to describe the amount of work performed by a professional. Although it is frequently used, it is often challenging to accurately measure each individual’s workload (22). A high workload is known to increase the risk of alcohol and drug abuse, depression and anxiety, and suicide in doctors (23). A Japanese study showed that high levels of work stress led to anxiety symptoms in nearly a third of physicians, with 20% affected by depressed mood (24). Studies have shown that stress is a risk factor that may lead to alcohol or other substance use (25, 26). People with a prior habit of alcohol use tend to drink more to cope with increased work pressures (27). Clinicians who work in psychiatry experience higher levels of occupational stress and are more likely to experience burnout (28). This is because of the specific nature of their work environment, the patient population they work with, and the need to manage stress levels for themselves and their team (29). A Turkish study showed that psychiatrists experience higher levels of work stress than doctors in other departments (30). A French study (302 interns in psychiatry and 1863 in other specialties) showed that interns in psychiatry had higher rates of alcohol use disorders than those in other specialties (31). A recent survey in China before the COVID-19 pandemic showed that Chinese psychiatrists’ workload and job burnout are increasing (32) and this trend likely worsened during the pandemic.

Currently, few studies have examined the relationship between workload and alcohol use or AUD among psychiatrists. Exploring the relationship between workload and alcohol consumption of this particular occupational group may provide insight into the mental health and their coping behaviors in response to stress and negative emotions. Considering previous research demonstrating that alcohol is commonly used to cope with stress, we hypothesize that increased workload among Chinese psychiatrists in turn increases the amount of alcohol consumed and the risks of developing AUD. To test this hypothesis, we conducted a survey study based on a nationally representative sample.

## 2. Methods

### 2.1. Study design, setting, and participants

This study was an online survey conducted in January 2021 and it was part of a large, national survey, the China National Healthcare Improvement Initiative Survey. We contacted all healthcare professionals working in 41 large public psychiatric tertiary hospitals in 29 provinces in China. All participants signed the informed consent before they proceeded to respond to the questionnaires. The informed consent statement explained the purpose of the survey, ensured that the data would be de-identified before analysis and that the hospitals’ administrators would have no access to their responses. In total, 21,858 psychiatrists, psychiatric nurses, and therapists participated. For this analysis, we included data from 3,549 psychiatrists. The survey was conducted through WeChat, an online social media platform, due to its widespread popularity and ease of use in China. To avoid repeated submissions, each WeChat account could only answer the questionnaire once. We collected information on sociodemographic data, workload, alcohol consumption, and other health-related behaviors. The Alcohol Use Disorder Identification Test-Consumption (AUDIT-C) was used to assess the frequency and amount of alcohol use.

### 2.2. Questionnaire design

The questionnaire was developed based on previous research experience and expert opinions. A pilot study was conducted to evaluate the feasibility of an online questionnaire before a formal questionnaire became available to all participants.

We collected socio-demographic data from all participants, including age, sex, education, and marital status. According to the World Health Organization standards, they were divided into three groups (20–34 years old, 35–49 years old, and ≥ 50 years old).

AUDIT-C is a simplified version of AUDIT but has been shown to have similar reliability to the full version of AUDIT (33–35). The score of the AUDIT-C questionnaire ranged from 0 to 12 points and is positively correlated with the severity of drinking. The Chinese version of AUDIT has been verified for validity and reliability (36). Detailed information about AUDIT-C scoring can be found elsewhere (14, 37–39). According to the AUDIT-C score, the respondents were divided into the no-alcohol use group (total score = 0) and the alcohol use group (total score ≥ 1). Based on the cutoff scores of the AUDIT-C (14), participants were further classified into probable alcohol misuse and non-alcohol misuse group (AUDIT-C score ≥ 3 for females and AUDIT-C score ≥ 4 for males).

In the workload section, based on previous studies and the working conditions of psychiatric medical institutions in China (22, 40), we collected information such as the working years, administrative position, working days per week, working hours per day, night shifts per month, outpatient visits per week, and the number of inpatients per day. Health-related lifestyle habits including physical activity, sleep, and smoking was also collected. “Regular exercise” was defined as exercising at least three times a week in the past month based on national fitness guidelines. “Frequent insomnia” was defined as having trouble sleeping (difficulty falling asleep, staying asleep, or waking up early) at least three times a week in the past month. Cigarette smoking was defined as having used more than 100 cigarettes in their lifetime (41).

### 2.3. Ethics statement

The study was approved by the Ethics Committee of Chaohu Hospital of Anhui Medical University (202002-KYXM-02). The survey was anonymous. Consent was obtained from participants when they accessed the online questionnaire.

### 2.4. Statistical analysis

In the first step, a single sample K-S test was used for statistical analysis to evaluate whether the continuous variables conform to the normal distribution. In the second step, ANOVA was used for continuous variables with normal distribution, and rank-sum tests were used for continuous variables out of normal distribution. In the third step, a single-factor analysis using the Chi-square test. OR values and 95% confidence intervals were calculated by binary logistic regression, and multivariate analysis was performed. All statistical analyses were performed using IBM Social Science Statistical Package (SPSS 23.0 edition), with a two-tailed *p* value of 0.05, indicating statistically significant differences. Regarding working hours, we used the cutoff defined by China’s Labor Law (42), which states the legal working hours per week should not exceed 44 h, therefore the participants were divided into overworked and non-overworked groups.

## 3. Results

### 3.1. Demographic and workload characteristics of participants

As shown in Table 1, the mean age of all participants was 38.2(±8.5) years old. Psychiatrists with bachelor degrees account for the largest proportion (64.4%), followed by those with additional master’s degrees (29.6%), and only 6.0% of psychiatrists have an additional doctorate. The average working time of all respondents was more than 5 days per week (5.6 ± 0.7), more than 8 h per day (8.9 ± 1.9), and more than 50 h per week (50.3 ± 13.6). Nearly two-thirds (64.4%) of psychiatrists worked more than 44 h a week on average, and more than one quarter (25.7%) worked more than 56 h a week. Nearly 60% (59.9%) worked at least three-night shifts a month, and more than a quarter (26.7%) worked at least five. The average number of outpatients per week was more than 50 (50.8 ± 64.2), 65% saw more than 15 outpatients per week, and 30.1% saw more than 50 outpatients per week. The average number of inpatients per day was 12.44 ± 8.12, 69.4% were in charge of 10 inpatients a day. 14.7% reported cigarette smoking (35.6% in males and 0.84% in females), 16.9% reported insomnia, and only 8.1% reported regular exercise.

**Table 1:** Basic features of psychiatrists, and subgroup comparisons between those who endorsed alcohol use and those who did not.

Variables	All participants (*n* = 3,549)	Alcohol use		*F* or *x*^2^	*p*-value
		No (*n* = 1860)	Yes (*n* = 1,689)		
Age	38.20 ± 8.52	37.58 ± 8.25	38.88 ± 8.76	20.696 ^a^	<0.001
*Age group (N, %)*					
20–34	1,374 (38.7%)	788 (57.4%)	586 (42.6%)	25.027^b^	<0.001
35–49	1713 (48.3%)	861 (50.3%)	852 (49.7%)		
≥50	462 (13.0%)	211 (45.7%)	251 (54.3%)		
*Sex (N, %)*					
Male	1,411 (39.8%)	365 (25.9%)	1,046 (74.1%)	661.497 ^b^	<0.001
Female	2,138 (60.2%)	1,495 (69.9%)	643 (30.1%)		
*Marital status (N, %)*					
Single	617 (17.4%)	305 (49.4%)	312 (50.6%)	8.893 ^b^	0.012
Married	2,800 (78.9%)	1,499 (53.5%)	1,301 (46.5%)		
Divorced or widowed	132 (3.7%)	56 (42.4%)	76 (57.6%)		
*Education (N, %)*					
Bachelor or below	2,287 (64.4%)	1,133 (49.5%)	1,154 (50.5%)	27.531^b^	<0.001
Master degree	1,049 (29.6%)	621 (59.2%)	428 (40.8%)		
Doctoral degree	213 (6.0%)	106 (49.8%)	107 (50.2%)		
*Geographical Region (N, %)*					
East China	1,398 (39.4%)	786 (56.2%)	612 (43.8%)	35.386 ^b^	<0.001
Central China	790 (22.3%)	438 (55.4%)	352 (44.6%)		
West China	988 (27.8%)	483 (48.9%)	505 (51.1%)		
Northeast China	373 (10.5%)	153 (41.0%)	220 (59.0%)		
Working hours per week	50.29 ± 13.59	49.89 ± 13.81	50.73 ± 13.34	3.368^a^	0.027
*Working hours per week*					
0 ~ 44	1,265 (35.6%)	711 (56.2%)	554 (43.8%)	11.358^b^	0.001
45~	2,284 (64.4%)	1,149 (50.3%)	1,135 (49.7%)		
*Night shifts per month*					
0 ~ 2	1,424 (40.1%)	735 (51.6%)	689 (48.4%)	1.857^b^	0.395
3 ~ 4	1,179 (33.2%)	637 (54.0%)	542 (46.0%)		
5~	946 (26.7%)	488 (51.6%)	458 (48.4%)		
*Outpatients per week*					
0 ~ 15	1,244 (35.0%)	672 (54.0%)	572 (46.0%)	4.121^b^	0.127
16 ~ 50	1,237 (34.9%)	655 (53.0%)	582 (47.0%)		
51~	1,068 (30.1%)	533 (49.9%)	535 (50.1%)		
*Inpatients per day*					
0 ~ 9	1,085 (30.6%)	549 (50.6%)	536 (49.4%)	3.262 ^b^	0.196
10 ~ 15	1,275 (35.9%)	692 (54.3%)	583 (45.7%)		
16~	1,189 (33.5%)	619 (52.1%)	570 (47.9%)		
*Administrative position*					
No	2,847 (80.2%)	1,589 (55.8%)	1,258 (44.2%)	66.866^b^	<0.001
Yes	702 (19.8%)	271 (38.6%)	431 (61.4%)		
*Smoking habit*					
No	3,029 (85.3%)	1768 (58.4%)	1,261 (41.6%)	294.415^b^	<0.001
Yes	520 (14.7%)	92 (17.7%)	428 (82.3%)		
*Frequent insomnia*					
No	2,950 (83.1%)	1,552 (52.6%)	1,398 (47.4%)	0.283^b^	0.595
Yes	599 (16.9%)	308 (51.4%)	291 (48.6%)		
*Regular exercise*					
No	3,262 (91.9%)	1722 (52.8%)	1,540 (47.2%)	2.342^b^	0.126
Yes	287 (8.1%)	138 (48.1%)	149 (51.9%)		

Frequent insomnia: having sleep disturbances at least three times per week; regular exercise: exercising at least three times per week.

a ANOVA.

b Chi-square test.

### 3.2. Alcohol use and misuse and related factors among psychiatrists

Male psychiatrists had significantly higher rates of alcohol use than female psychiatrists (74.1% vs. 30.1%, *p* < 0.001). The average age of the drinking group was higher than that of the non-drinking group (38.9 ± 8.8 vs. 37.6 ± 8.3, *p* < 0.001), and the rate of alcohol use increased with age. Compared with married psychiatrists, single, divorced or widowed psychiatrists had significantly higher rates of alcohol use (50.6, 57.6% vs. 46.5%, *p* = 0.012). Psychiatrists with additional master’s degrees had the lowest rates of alcohol use compared to those with college (medical degree), or those with an additional doctoral degree (40.8% vs. 50.5, 50.2%, *p* < 0.001). Psychiatrists in the Northeast region had higher rates of alcohol use than those in other geographical regions. Psychiatrists who reported alcohol use worked significantly longer hours per week than those who did not (50.73 ± 13.34 vs. 49.89 ± 13.81, *p* = 0.027). Psychiatrists who worked 44 h or less per week had significantly lower rates of alcohol use than those who worked more than 44 h per week (43.8% vs. 49.7%, *p* = 0.001). Alcohol consumption among psychiatrists with administrative positions was significantly higher than that of psychiatrists without administrative positions (61.4% vs. 44.2%, *p* < 0.001). The rate of alcohol use in psychiatrists who reported smoking nearly doubled that in those who did not smoke (82.3% vs. 41.6%, *p* < 0.001). There were no statistically significant associations between the number of night shifts per month or the number of inpatients cared for per day and alcohol use.

Using the AUDIT-C cutoff scores, 287 psychiatrists (8.1%) were classified as having probable alcohol misuse (or AUD) and the rate of alcohol misuse in males (19.6%) was more than 7 times higher than that in female psychiatrists (2.6%, *p* < 0.001). The average age of the alcohol misuse group was higher than that of the non-alcohol misuse group (40.17 ± 8.93 vs. 38.02 ± 8.46, *p* < 0.001), and the rate of alcohol misuse increased with age, from 5.7% in the low age group (20–34 years old) to 9.0% in the middle and high age group (35–49 years old) and 11.5% in the ≥50 years old group. Psychiatrists in the Northeast region had higher rates of alcohol misuse than those in other geographical regions. The average number of night shifts per month of the alcohol misuse group was lower than that of the non-alcohol misuse group (2.77 ± 2.51 vs. 3.21 ± 2.64, *p* < 0.001), and the rate of alcohol misuse decreased with the number of night shifts per month, from 10.0% in the low night shifts group (0-2night shifts per month) to 7.3% in the middle and high night shifts group (3–4 night shifts per month) and 6.2% in the ≥5 night shifts group. Alcohol misuse among psychiatrists with administrative positions was significantly higher than that of psychiatrists without administrative positions (13.5% vs. 6.7%, *p* < 0.001). Psychiatrists who reported smoking had a significantly higher rate of alcohol misuse than psychiatrists who did not smoke (27.1% vs. 4.8%, p < 0.001). Psychiatrists who reported frequent insomnia had a significantly higher rate of alcohol misuse than psychiatrists who did not (12.5% vs. 7.2%, p < 0.001).

### 3.3. Correlation between AUDIT-C scores and workload

The AUDIT-C total score ranges from 0 to 12, with higher scores indicating elevated risk for hazardous drinking or AUD. As shown in Table 2, in spearman correlation analysis, AUDIT-C scores were positively correlated with working hours per week (Rs = 0.040, *p* = 0.017) and outpatients per week (Rs = 0.046, *p* = 0.006). However, there was no statistically significant relationship between the number of night shifts per month or the number of inpatients cared for per day and AUDIT-C scores.

**Table 2:** Spearman correlation analysis of workload and AUDIT-C scores.

	AUDIT-C scores	
	Rs	*p*-value
Working hours per week	0.040	0.017*
Night shifts per month	−0.009	0.597
Outpatients per week	0.046	0.006**
Inpatients per day	−0.018	0.286

The AUDIT-C total score ranges from 0 to 12, with higher scores indicating more severe drinking.

** = statistically significant at *p*<0.01 level; * = statistically significant at *p*<0.05 level.

### 3.4. Factors associated with alcohol use in a binary logistic regression

As shown in Table 3, we used binary logistic regression to examine factors associated with alcohol use among psychiatrists who drank alcohol and those who did not. Psychiatrists who worked more than 44 h per week had a significantly increased risk of alcohol use compared to those who worked 44 h or less per week (OR = 1.315; 95% CI =1.115–1.551). Psychiatrists with administrative positions (OR = 1.498; 95%CI =1.207–1.858) have a higher risk of alcohol use than psychiatrists without administrative positions. Male (OR = 6.856; 95%CI =5.888–7.983), single (OR = 1.601; 95%CI =1.282–1.998), divorced or widowed (OR = 1.888; 95%CI =1.274–2.799), smoking habit (OR = 2.219; 95%CI =1.699–2.899), working in the western region (OR = 1.511; 95%CI =1.240–1.840), work in Northeast China (OR = 2.440; 95%CI =1.833–3.247) were risk factors for alcohol use. Being female, being married, and not smoking were protective factors for alcohol use among psychiatrists. However, there was no statistically significant relationship comparing the number of night shifts, the number of inpatients, the number of outpatients, and whether or not they consumed alcohol.

**Table 3:** Logistic regression examining individual characteristics associated with alcohol use in psychiatrists in China.

Variables	OR	95% CI	*p*-value
*Hours worked per week (ref. 0 ~ 44)*			
45~	1.315	1.115–1.551	0.001
*Night shifts per month (ref. 0 ~ 2)*			
3 ~ 4	0.936	0.731–1.199	0.602
5~	0.950	0.650–1.391	0.793
*Outpatients per week (ref. 0 ~ 15)*			
16 ~ 50	0.994	0.813–1.215	0.950
51~	0.958	0.705–1.302	0.783
*Inpatients per day (ref. 0 ~ 9)*			
10 ~ 15	0.942	0.699–1.270	0.695
16~	1.017	0.634–1.630	0.944
*Administrative position (ref. No)*			
Yes	1.498	1.207–1.858	<0.001
*Sex (ref. Female)*			
Male	6.856	5.888–7.983	<0.001
*Marital statu s(ref. Married)*			
Single	1.601	1.282–1.998	<0.001
Divorced or widowed	1.888	1.274–2.799	0.002
*Geographical region (ref. East China)*			
Central China	1.019	0.826–1.257	0.862
West China	1.511	1.240–1.840	<0.001
Northeast China	2.440	1.833–3.247	<0.001
*Smoking habit (ref. No)*			
Yes	2.219	1.699–2.899	<0.001

### 3.5. Factors associated with alcohol misuse in a binary logistic regression

Furthermore, using the AUDIT-C cutoff, we run a similar logistic analysis to explore factors associated with probable alcohol misuse, and we found psychiatrists who worked fewer than 2 night shifts per month had significantly higher rates of alcohol misuse than those who worked more than 5 night shifts per month (OR = 1.864; 95% CI =1.271–2.735). Psychiatrists who worked 3 to 4 night shifts per month had significantly higher rates of alcohol misuse than those who worked more than 5 night shifts per month (OR = 1.460; 95% CI =1.006–2.119). Male (OR = 4.007; 95%CI =2.837–5.659), working in Northeast China (OR = 1.683; 95%CI =1.098–2.580), smoking habit (OR = 3.199; 95%CI =2.370–4.317), frequent insomnia (OR = 1.678; 95%CI =1.230–2.290) were risk factors for alcohol misuse. Being female, good sleep quality, and not smoking were protective factors for alcohol misuse among psychiatrists. However, there was no statistically significant relationship comparing the number of hours worked per week, the number of inpatients, the number of outpatients, and whether or not they are alcohol misusers (Table 4).

**Table 4:** Logistic regression examining individual characteristics associated with alcohol misuse in psychiatrists in China.

Variables	OR	95% CI	*p*-value
*Hours worked per week (ref. 0 ~ 44)*			
45~	1.107	0.826–1.485	0.495
*Night shifts per month (ref. 5~)*			
3 ~ 4	1.460	1.006–2.119	0.046
0 ~ 2	1.864	1.271–2.735	0.001
*Outpatients per week (ref. 0 ~ 15)*			
16 ~ 50	1.112	0.790–1.565	0.543
51~	1.190	0.838–1.691	0.331
*Inpatients per day (ref. 0 ~ 9)*			
10 ~ 15	0.745	0.529–1.051	0.094
16~	0.949	0.678–1.328	0.760
*Administrative position (ref. No)*			
Yes	1.278	0.918–1.777	0.146
Sex (ref. Female)			
Male	4.007	2.837–5.659	<0.001
*Geographical region (ref. East China)*			
Central China	1.382	0.967–1.975	0.076
West China	1.388	0.976–1.973	0.068
Northeast China	1.683	1.098–2.580	0.017
*Smoking habit (ref. No)*			
Yes	3.199	2.370–4.317	<0.001
*Frequent insomnia (ref. No)*			
Yes	1.678	1.230–2.290	0.001

## 4. Discussion

This is the first large-scale study to investigate the association between workload-related factors and alcohol use and alcohol misuse among psychiatrists in China. The sample accounted for 85.6% of all psychiatrists working in those top tier psychiatric hospitals. They may not represent psychiatrists working in secondary or rural psychiatric settings. We found that Chinese psychiatrists often work long hours (often exceeding the legal limit) with multiple nightshifts and large caseloads. We also found nearly half (47.6%) of psychiatrists in China reported alcohol use and 8.1% had probable alcohol misuse. We found that workload, including the working hours per week, number of outpatients, and administrative positions was significantly associated with a risk of alcohol use. We found that workload, including the number of night shifts per month, number of outpatients, and administrative positions was significantly associated with a risk of alcohol misuse. We found that psychiatrists who were male, single, divorced or widowed, worked in Western or Northeastern China, and those who had a smoking habit had a higher risk of alcohol use. We also found that alcohol misuse in psychiatrists was significantly associated with the following factors: being male, working in Northeastern China, smoking habit, and reporting frequent insomnia.

### 4.1. Alcohol use and misuse and workload among Chinese psychiatrists

In this study, we found overwork was common in this sample. Psychiatrists in our sample nearly worked 6 days a week and 9 h per day. Nearly two thirds worked more than 44 h a week, the limit set by the China’ Labor Law. These heavy workloads are concerning as they likely affect their work-life balance, contribute to burnout and other psychological issues. Similar situations were found in other countries. For example, a 2020 study from Thailand suggested that more than 80% of Thai psychiatrists worked >40 h per week, including 33.6% who worked >50 h per week, with long work hours associated with high burnout. The study also suggests that the more patients per day, the more work stress (43). A 2010 study indicated that work stress was most closely linked to long working hours (44). A 2016 long-term follow-up study from Europe showed that working long hours can lead to higher job stress and burnout (45). Psychiatrists work long hours and excessive workloads, which can lead to higher occupation-related stress. Occupational stress is defined as the stress caused by work, which can have a series of adverse effects on staff’s physical and mental health (30). The current COVID-19 pandemic has increased the stress level of society in general, including the psychiatric community, and the necessary restrictions will lead to an economic downturn in society. They will also bring additional mental health distress (46). Good mental health services depend on psychiatrists investing significant time and effort. At the same time, due to the unique nature of their profession, physicians are generally unable to admit that they are facing stress and psychological problems and are unlikely to seek help. They often sacrifice personal time, benefits, and family responsibilities so they can continue to devote themselves to their work (47, 48). A Turkish study showed that psychiatrists are under more pressure than other medical specialists (30). Systematic interventions are needed to relieve their work stress and thus promote their physical and mental health (30). An Australian study revealed that doctors have higher rates of substance misuse, including alcohol misuse, due to work stress than the general population (49). At the same time, studies have found that psychiatrists have higher job stress and burnout than other physicians (50). In 2013, a U.S. study showed higher rates of alcohol use and misuse among physicians compared with general controls (51). Subsequently, many psychiatrists feel physically and mentally tired and turn to drinking to cope (52).

This study showed that in China, psychiatrists who worked more than 44 h per week had a significantly higher risk of alcohol use than those who worked 44 h or less per week (OR = 1.315, *p* = 0.001). This suggests that patient workload among psychiatrists is associated with alcohol consumption. Two studies from Taiwan and mainland China, were consistent with our findings that longer working hours were associated with a higher risk of drinking and alcohol misuse (53, 54). A 2008 Canadian study also achieved results consistent with ours (55). The study also found a clear correlation between the number of outpatients psychiatrists saw and the risk of alcohol use. The greater the number of outpatient visits and the busier the outpatient workload, the higher the risk of alcohol use. A 2012 foreign study agrees that heavy work stress, including outpatient work stress, increases the risk of alcohol use among doctors, especially younger ones (56). Outpatient psychiatrists carry the burden of heavy patient loads, are required to be attentive for extended periods of time, and deal with a significant amount of the negative effects of their patients.

It is imperative to protect psychiatrists from high-intensity work pressure and reduce the adverse effects of an overburdened workload. Reducing work stress and working hours can lower the risk of alcohol use in psychiatrists. Systematic measures and individual self-regulation are the basis for improving occupational health (57). These measures could include ongoing monitoring of employee health and providing burnout/stress reduction training to optimize individual coping strategies and providing professional supervision groups and personal counseling services. Providing more resources and personnel is the most basic measure to reduce workload (58, 59).

### 4.2. Administrative work and alcohol use

Studies suggest that job position may play a role in the risk of using or misusing alcohol. Workers in management positions are at higher risk of alcohol use and abuse (55). Similar trends are found in medical institutions. Career development for psychiatrists may include taking on an administrative position. Our study suggests that 19.8% of Chinese psychiatrists perform medical and administrative duties. The risk of alcohol use was significantly higher among psychiatrists with administrative positions than among those without administrative positions (61.4% vs. 44.2%, *p* < 0.001); psychiatrists with administrative positions had a significantly higher risk of alcohol misuse than those without administrative positions (13.5% vs. 6.7%, *p* < 0.001). Our findings are consistent with many previous studies showing that psychiatrists who have been working longer in the field and those in management positions have a higher risk of alcohol use and misuse than younger staff in their units (60–62).

The results of our study fit well with our current work and social context. Psychiatrists who are older and with more working experience are more likely to be in administrative positions and have more opportunities to participate in social interaction activities. In China’s social interaction environment, participating in social interaction activities often requires drinking, so psychiatrists with administrative positions have a higher risk of alcohol use.

### 4.3. Demographics and alcohol use or misuse

Our study found that male psychiatrists in China have a significantly higher risk of alcohol use and misuse than female psychiatrists, consistent with several previous studies in different countries and regions (6, 9, 14, 63). In Chinese culture, it is generally frowned upon for women to drink, and women who smoke and drink may be discriminated against. Among Chinese psychiatrists, our study also found a gradual increase in the risk of alcohol use and misuse with increasing age, which is consistent with previous findings in China (14, 37). Most studies abroad have found the same results as us, namely that the risk of alcohol use and misuse increases with age (9, 25, 63).

Our study found a significant difference in the risk of alcohol use among psychiatrists of different marital statuses in China. Married psychiatrists had the lowest risk of alcohol use, while divorced psychiatrists had the highest alcohol use. The results align with previous studies in China (4, 14, 37). Similar studies abroad have found that good marital relationships can reduce the risk of alcohol use and misuse, and alcohol use will increase significantly as marital relationships deteriorate and marriages break up (64, 65). Other studies have suggested that the relationship between alcohol use and marital status is complex, that they influence each other, and that it is not entirely possible to conclude that moving from single to married will necessarily reduce alcohol use (66). A good marital relationship and family atmosphere may help individuals cope better with their negative emotions and thus reduce the need to consume alcohol for self-medication.

### 4.4. Lifestyle and alcohol use

Our study found that alcohol use and misuse is closely related to region and lifestyle. Psychiatrists in northeast China had significantly higher rates of alcohol consumption than those in other regions, while the risk of alcohol use and misuse among psychiatrists in eastern China was the lowest, consistent with the results of previous domestic studies (14, 37). Because the northeast region is the coldest in China, the culture of drinking heavy liquor has been around since ancient times. Drinking heavy liquor can make people feel warm and resist cold. In this study, smoking led to a higher risk of alcohol use and misuse, which is consistent with the results of many previous studies in mainland China and Taiwan (4, 14, 37, 54). In China, cigarette and alcohol use are thought to be closely linked, and many people smoke while drinking. Many of the same studies abroad have reported that tobacco and alcohol use often co-occur (67). Some foreign studies have even proved that the risk of alcohol use is three times higher in regular smokers than in non-smokers. Smoking status is a good predictor of alcohol use and can be included in screening for alcohol use. However, the mechanism by which smoking increases alcohol use and misuse is very complex and likely has both biological and psychological underpinnings (68).

The study also found that psychiatrists with frequent insomnia had a significantly higher risk of alcohol misuse than those without insomnia, consistent with the findings of several previous national studies (14, 32, 37). Many international studies have confirmed that problems such as heavy drinking and alcohol use-related disorders appear to be bi-directional to sleep-related issues, such as insomnia and abnormal sleep rhythms. Animal studies have reached similar conclusions (51, 69, 70). Many patients with alcohol use-related disorders initially drink to improve sleep, but later, with increased use, insomnia can worsen. In the past, some researchers believed that drinking caused insomnia, while others tended to believe that insomnia caused the aggravation of the drinking problem. Now researchers at home and abroad have concluded that insomnia problems and drinking mutually influence rather than a unilateral causal relationship.

### 4.5. Limitations

There are some limitations to the study. First, we used a self-rated screening tool to assess alcohol use rather than provider-administered surveys or lab values, and thus results should be treated with caution. The cut-off point for “alcohol use and misuse” was based on other research in China and globally. Second, our participant pool only include psychiatrists in large public psychiatric institutions in big cities in China and excluded psychiatric hospitals in small towns or rural areas. However, the workload of doctors in large public psychiatric institutions in big cities is significantly different from that of doctors in small hospitals. Therefore, the study did not include all psychiatric groups and smaller psychiatric medical institutions in rural cities. Third, we collected various data points at different time ranges. For alcohol use, we assess past-year consumption. Meanwhile, we assessed exercise, insomnia, and outpatient and ward workload over the past month. These disparate time durations may influence our study results. Finally, many work-related factors that may modify work stress perception were not included in the study, such as workplace justice, job insecurity, workplace violence, and organizational alcohol management policies. Future studies need to consider these limitations.

## 5. Conclusion

As predicted, we found clear associations between alcohol use or misuse and heavier workload, which includes longer working hours, greater outpatient caseload, and combined administrative work. Contrary to our prediction, psychiatrists who worked more night shifts per month had lower rates of alcohol misuse. Being male, single, divorced, and living in the West and Northeast were also strongly associated with an increased risk of alcohol consumption. Being male, and working in the Northeast were strongly associated with an increased risk of alcohol misuse. Regarding lifestyle habits, psychiatrists who reported smoking had a significantly increased risk of alcohol use and psychiatrists who reported smoking and frequent insomnia had a significantly increased risk of alcohol misuse. Our study provides insights into the risk factors for alcohol use and misuse in this occupational group and suggests the need for policy change and additional support systems.

## Data availability statement

The raw data supporting the conclusions of this article will be made available by the authors, without undue reservation.

## Ethics statement

The studies involving human participants were reviewed and approved by the Ethics Committee of Chaohu Hospital of Anhui Medical University (202002-KYXM-02). The patients/participants provided their written informed consent to participate in this study.

## Author contributions

FJ, HL, and Y-lT: study design. WL, LC, DM, LX, and Y-lT: collection, analyses, and interpretation of data. WL: drafting the first version of the manuscript. MH and Y-lT: critical revision of the manuscript. All authors contributed to the article and approved the submitted version.

## Funding

The study was supported by the National Clinical Key Specialty Project Foundation (CN), and the Beijing Medical and Health Foundation (Grant No. MH180924).

## Conflict of interest

The authors declare that the research was conducted in the absence of any commercial or financial relationships that could be construed as a potential conflict of interest.

## Publisher’s note

All claims expressed in this article are solely those of the authors and do not necessarily represent those of their affiliated organizations, or those of the publisher, the editors and the reviewers. Any product that may be evaluated in this article, or claim that may be made by its manufacturer, is not guaranteed or endorsed by the publisher.
